# Silver nanoparticles selectively induce human oncogenic γ-herpesvirus-related cancer cell death through reactivating viral lytic replication

**DOI:** 10.1038/s41419-019-1624-z

**Published:** 2019-05-21

**Authors:** Chunlei Wan, Jiahui Tai, Jie Zhang, Yi Guo, Qing Zhu, Ding Ling, Feng Gu, Jin Gan, Caixia Zhu, Yuyan Wang, Sijin Liu, Fang Wei, Qiliang Cai

**Affiliations:** 1 MOE&NHC&CAMS Key Laboratory of Medical Molecular Virology, School of Basic Medical Science, Shanghai Medical College, Fudan University, Shanghai, 200032 P. R. China; 2 State Key Laboratory of Environmental Chemistry and Ecotoxcicology, Research Center for Eco-Environmental Science, Chinese Academy of Sciences, Beijing, 100085 P. R. China; 3 Department of Gynecology, Key laboratory of AIDS immunology of Ministry of Health, First Affiliated Hospital of China Medical University, Shengyang, 110000 P. R. China; 4 ShengYushou Center of Cell Biology and Immunology, School of Life Sciences and Biotechnology, Shanghai Jiao Tong University, Shanghai, 200240 P. R. China; 5 Expert Workstation, Baoji Central Hospital, Baoji, 721008 P. R. China

**Keywords:** Drug development, Tumour virus infections

## Abstract

Silver nanoparticle (nAg), which is one of the most common manufactured nanomaterials, has a wide range of biomedical applications. The human oncogenic γ-herpesviruses, Kaposi’s sarcoma-associated herpesvirus (KSHV) and Epstein–Barr Virus (EBV), are etiologically linked to many malignancies. Currently, there are no efficient or specific treatments for these types of tumors, and most patients die because of resistance to conventional cytotoxic chemotherapy. Despite nAg having antitumor and antiviral activities, its effects on oncogenic herpesvirus-related cancer cells remain largely unknown. Here, we reveal that nAg presents higher cytotoxicity against KSHV- or EBV-latently infected cells via reactivating viral lytic replication, which relies on the induction of reactive oxygen species (ROS) generation and autophagy. Moreover, nAg blocks KSHV primary infection by directly destroying virion particles, as well as effectively inhibits colony formation and moderately represses the growth of KSHV-associated tumors in xenograft mouse model. Taken together, these results demonstrate the therapeutic potential of nAg for use in the antiviral infection and treatment of oncogenic herpesvirus-related cancers.

## Introduction

Silver nanoparticle (nAg) is an emerging material with a high surface area to volume ratio and unique physico-chemical properties, because of its nanoscale that has shown a wide range of attractive biomedical application^1^. In the antiviral field, some studies have demonstrated the remarkable antiviral ability of nAg toward several viruses including human immunodeficiency virus (HIV)^2–4^, hepatitis B virus (HBV)^5^, herpes simplex virus (HSV)^6,7^, and influenza virus^8^. Due to the complicated steps for each viral infection, the detail mechanism of nAg against a specific viral infection could be different. For examples, the potential mechanisms of the antiviral activity of nAg could be relied on either interaction with viral surface glycoproteins, competition for binding of the virus to host cells, inactivation of viral particles prior to entry, or impairing viral double-stranded DNA. However, distinct from conventional antiviral drugs, nAg can attack different viruses and a broad range of viral components, which can reduce the possibility of drug resistance. In the anticancer field, some studies have also demonstrated that nAg displays anticancer activity, particularly higher sensitivities of cancer cells than normal cells to nAg^9–11^. Importantly, some reports showed that nAg could not only prevent vascular endothelial growth factor-induced (VEGF) or fibroblast growth factor-2 (FGF-2)-induced angiogenesis, which is crucial for growth and spread of tumors^12,13^, but that it also significantly inhibited tumor growth in animal models^14,15^. However, in regard to those cancers that are associated with oncogenic virus infection, the role of nAg on viral-related cancer cells remains unclear.

Human oncogenic γ-herpesviruses are enveloped icosahedral double-stranded DNA viruses that include Kaposi’s sarcoma-associated herpesvirus (KSHV) and Epstein–Barr virus (EBV)^16^. These two γ-herpesviruses, which have been shown to infect different cell types including B cells, endothelial cells, and epithelial cells, are closely associated with many human malignancies. EBV, the first human tumor virus discovered in 1964, causes more than 200,000 cases of cancer every year^17^. This virus could not only primarily cause various lymphomas, including Burkitt lymphoma (BL), Hodgkins lymphoma (HL), NK-T cell lymphoma and immunodeficiency associated lymphoma, but also induce some carcinomas, such as nasopharyngeal carcinoma (NPC) and EBV-associated gastric carcinoma (EBV-GC)^18^. While KSHV was originally identified from Kaposi’s sarcoma (KS) tissues in an AIDS patient in 1994 by Chang and Moore, and it is also associated with various lymphomas, including primary effusion lymphoma (PEL), Multicentric Castleman’s disease (MCD) and KSHV-associated germinotropic lymphoproliferative disorder^19^. These diseases often occur in populations subject to immunosuppression, and the infection rates of KSHV are dependent on geographic location^20^. Like other herpesviruses, the life cycle of KSHV and EBV comprises latency and lytic replication. It has been demonstrated that KSHV/EBV-associated tumors usually harbor latent infection with limited expression of latent genes, which is closely related to tumor development, and that a few cells with spontaneous lytic replication promote viral spread and disease maintenance. To date, no standard treatments for KSHV/EBV-associated malignancies have been developed, and current treatments are suboptimal and primarily associated with toxicity. Nonspecific chemotherapeutic drugs are common clinical therapies for malignancies with KSHV/EBV infection, which usually cause considerable side effects. Considering the role of KSHV/EBV in tumors, many studies have focused on the small molecules specifically targeting viruses^21^. Many small molecules have been shown to either inhibit lytic replication or block the transmission of KSHV/EBV from cell to cell (which cannot clear the virus lurking in cells), or to effectively induce viral lytic replication and promote tumor cell death and exposure of the virus to host immune surveillance^21^. However, given that both lytic replication and constant infection of new cells contribute to viral propagation and viral pathogenesis^22^, agents targeting both lytic replication and viral primary infection could be promising drugs for use in treatment of KSHV/EBV-related cancers.

In this study, we explored the effects of nAg on KSHV/EBV-associated tumor cells in vitro and in vivo. We identified spherical nAg with a size of 25 nm as an appropriate nanoparticle for induction of tumor cell cytotoxicity in a dose- and shape-dependent manner. Moreover, we demonstrated that the 25 nm-spherical nAg-induced greater cytotoxicity in KSHV/EBV-latently infected cells through reactivating viral lytic replication, which is relied on induction of reactive oxygen species (ROS) generation and autophagy. Importantly, the nAg not only blocks KSHV primary infection by directly destroying virion particles, but it also effectively inhibits colony formation and moderately represses the growth of KSHV-associated PEL tumors in xenograft mouse model. This evidence suggests that nAg could be a potential agent for treating the development of oncogenic herpesvirus-associated cancers.

## Results

### nAg particles exert size- and shape-dependent cytotoxicity

**Fig. 1:**
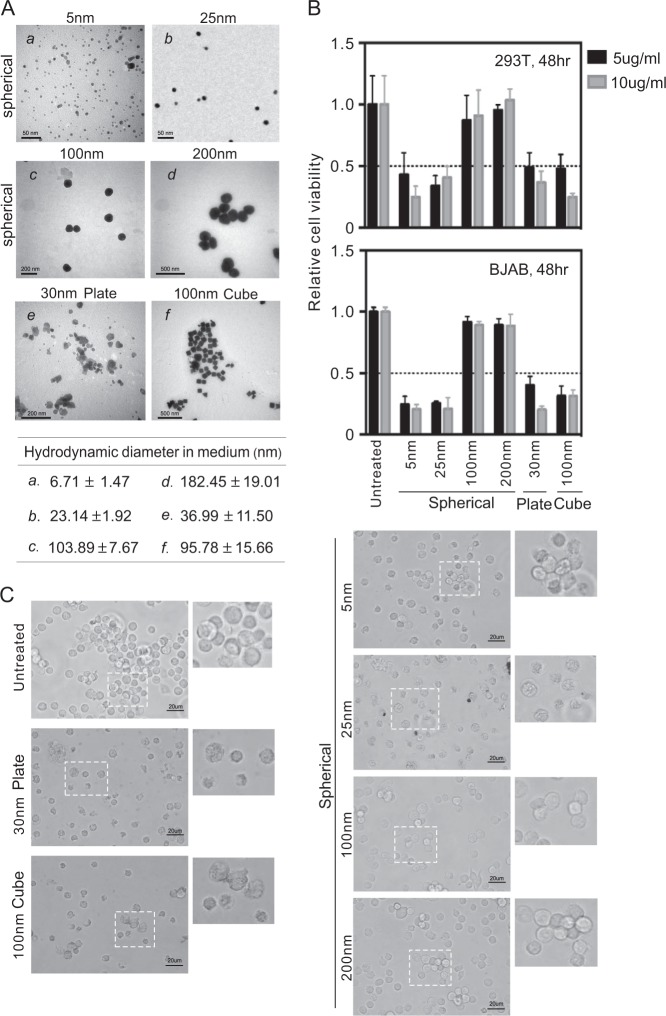
nAg particles present size- and shape-dependent cytotoxicity. **a** Representative photograph of nAg with different size and shape under TEM. Scale bars are 50, 200, and 500 nm as indicated in the figure. The hydrodynamic diameter of each nAg in tissue culture medium was shown at the bottom panel. **b** Relative viability of 293 T and BJAB cells after incubation with nAg of different size and shape for 48 h. The data shown are the means ± SD from triplicate analyses. **c** The morphology of BJAB cells after 10 μg/ml nAg treatment for 48 h. The magnificent photograph was shown on the right panel

As shown in Fig. 1a, transmission electronic microscope (TEM) was used to visualize the morphology of each polyvionylpyrolidone (PVP)-coated nAgs. To better mimic the condition in culture medium, the hydrodynamic diameter of each nAg in medium was also measured (Fig. 1a, bottom panel). To determine the characteristic of each PVP-coated nAg with different sizes and shapes, 293T and BJAB cells were individually used as models of the epithelium and B-lymphocyte to assess the cytotoxicity. As shown in Fig. 1b, the inhibitory effects of nAg on the viability of 293T cells were consistent with those on BJAB cells. Among spherical shaped particles, the smaller nAg (5, 25 nm) had a stronger inhibitory effect on cell viability than the larger nAg (100, 200 nm). At a size of 100 nm, the cytotoxicity of cubic nAg was greater than that of spherical nAg, which may have been because of the larger surface area (Fig. 1b). Morphological evaluation of the BJAB cells revealed that cells treated with 5 or 25 nm spherical nAg, 30 nm plate nAg or 100 nm cubic nAg became small and shrunken, while no significant changes were observed in cells treated with 100 or 200 nm spherical nAg (Fig. 1c). These findings further confirm that the cytotoxicity of nAg occurs in a size- and shape-dependent manner.

### nAg induces higher cytotoxicity in KSHV/EBV-latently infected cells

**Fig. 2:**
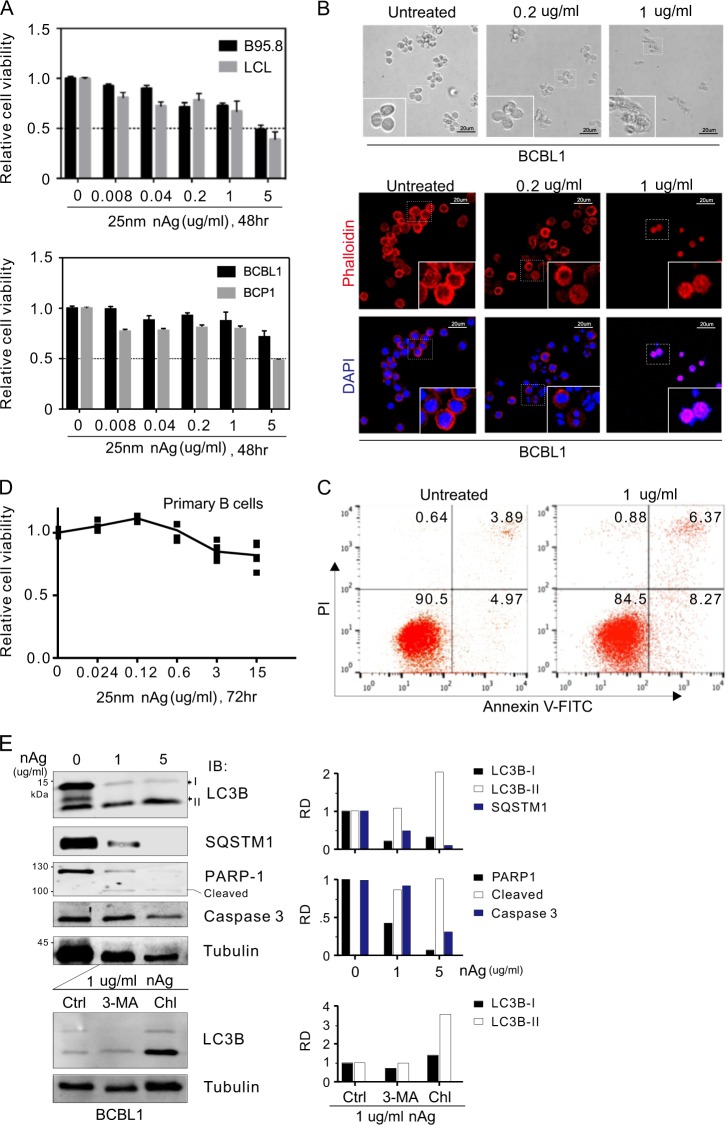
nAg induces KSHV/EBV-infected cell apoptosis. **a** Relative viability of KSHV/EBV-infected B-lymphoma cells after 25 nm-spherical nAg treatment. EBV-positive (B95.8, LCL) and KSHV-positive (BCBL1, BCP1) cells were subjected to cell viability assays during treatment with 25 nm-spherical nAg for 48 h. **b** Representative morphology of BCBL1 cells treated with nAg from **a** under the light microscope (top panel) and immunofluorescence microscope after TRITC-phalloidin staining (bottom panel). Nuclei are stained by DAPI. **c** nAg induces KSHV-infected cell apoptosis. BCBL1 cells untreated or treated with nAg (1 μg/ml) were subjected to flow cytometry analysis with Annexin V and PI staining. The percentage of cell population was shown in the figure. **d** Relative cell viability of primary B cells from human peripheral blood mononuclear cells (PBMCs) treated with different concentrations of 25nm-spherical nAg for 72 h. **e** Immunoblotting assays of LC3B, SQSTM1, PARP-1, and Caspase 3 in the BCBL1 cells treated with or without 25 nm-spherical nAg from **a**. At 24 h post-treatment with 1 μg/ml nAg, cells were individually subjected to treatment with lysosomal inhibitor Choloquine (Chl, 25 μM) or autophagy inhibitor 3-Methyladenine (3-MA, 10 mM) for 1 h, followed by immunoblotting with antibodies as indicated in the figure. Relative density (RD) of protein bands were quantified and shown on the right panel. Tubulin was used as a control

Previous studies showed that spherical nAg has significant effects and applications in many fields and can cross the plasma membrane more easily than other shapes^23^, and small sized nAg also tends to have a greater dissolution rate than large-sized nAg^23,24^. Therefore, we selected 25 nm-spherical nAg for subsequent experiments. To further evaluate the inhibitory effects of 25 nm-spherical nAg on the viral-infected B cells, different concentrations of nAg were used to treat KSHV-positive (BCBL-1, BCP1) and EBV-positive (B95.8, LCL) B-lymphoma cells for 48 h. The results of a cell viability assay and morphological observation of the cytoskeleton and apoptosis showed that the nAg decreased cell viability in a dose-dependent manner (Fig. 2a–c). In contrast, a weaker effect was observed on primary B cells from human PBMCs, for which the relative cell viability was always higher than 80%, even when it was incubated with 15 μg/ml nAg for 72 h (Fig. 2d), while the relative cell viability of KSHV/EBV-infected B-lymphoma cells generally decreased to <50% when treated with 5 μg/ml nAg for 48 h. Overall, these results suggest that KSHV/EBV-latently infected B-lymphoma cells were more sensitive to nAg than primary normal B cells.

Given that many signal pathways, including autophagy activation and apoptosis, are involved in cytotoxicity and that nAg has been reported to induce autophagy and apoptosis^25,26^, the active markers of LC3B-II and SQSTM1 of autophagy and cleaved PARP-1 and caspase 3 of apoptosis were examined in BCBL1 cells with different dosage of nAg treatment. The results showed that increase of LC3B-II accumulation and PARP-1 cleavage along with decrease of SQSTM1 and caspase 3 appeared dramatically in a dose-dependent manner when cells treated with nAg (Fig. 2e, top panels), indicating that nAg could induce stronger autophagy and apoptosis in KSHV-infected cells. In addition, the results of chloroquine (lysosome inhibitor) instead of 3-MA (inhibitor of LC3B-I to II switch) treatment resulted in led to further accumulation of LC3B-II in nAg-treated cells (Fig. 2e, bottom panels), confirming that nAg indeed induces autophagy in KSHV-infected cells.

**Fig. 3:**
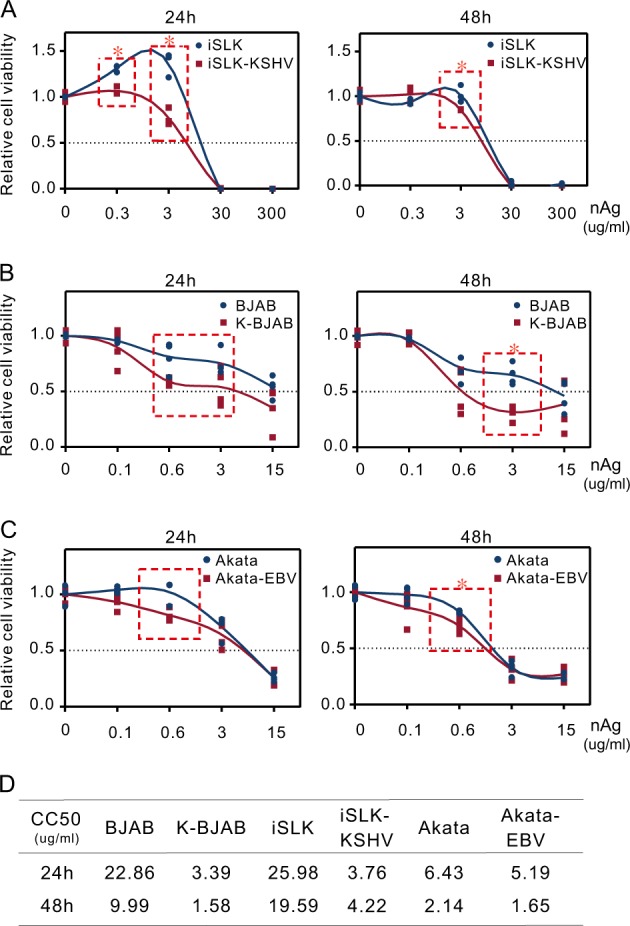
nAg presents higher cytotoxicity to KSHV/EBV-infected cells. Relative viability of **a** iSLK and iSLK-KSHV, **b** BJAB and K-BJAB, and **c** Akata and Akata-EBV cells after incubation with 25 nm-spherical nAg for 24 h or 48 h. The data are presented as the means ± SD from triplicate analyses. Asterisks indicate *P* value as follows: *P < 0.05. **d** The CC_50_ of iSLK, iSLK-KSHV, BJAB, K-BJAB, Akata and Akata-EBV cells from **a**, **b** and **c**

To further determine if nAg does have different effects on KSHV/EBV-positive and -negative tumor cells, three pairs of cell lines BJAB, iSLK and Akata with or without KSHV/EBV latent infection were individually incubated with nAg at different concentrations for 24 and 48 h, after which cell viability was measured and the 50% cytotoxic concentration (CC_50_) of each cell line was calculated. Interestingly, the CC_50_ values for KSHV/EBV-positive (K-BJAB, iSLK-KSHV, and Akata-EBV) cells were always consistently lower than those for KSHV/EBV-negative (BJAB, iSLK, and Akata) cells (Fig. 3a–d). Further statistical analyses showed that nAg had significantly greater inhibitory effects against KSHV/EBV-positive cells at an appropriate concentration (3 μg/ml for BJAB/K-BJAB and iSLK/iSLK-KSHV, 0.6 μg/ml for Akata/Akata-EBV), while no significant difference were observed under the too low or too high concentrations of nAg (Fig. 3a–c). These results suggest that KSHV/EBV-latently infected cells are indeed more vulnerable to nAg than KSHV/EBV-uninfected cells within a certain concentration range.

**Fig. 4:**
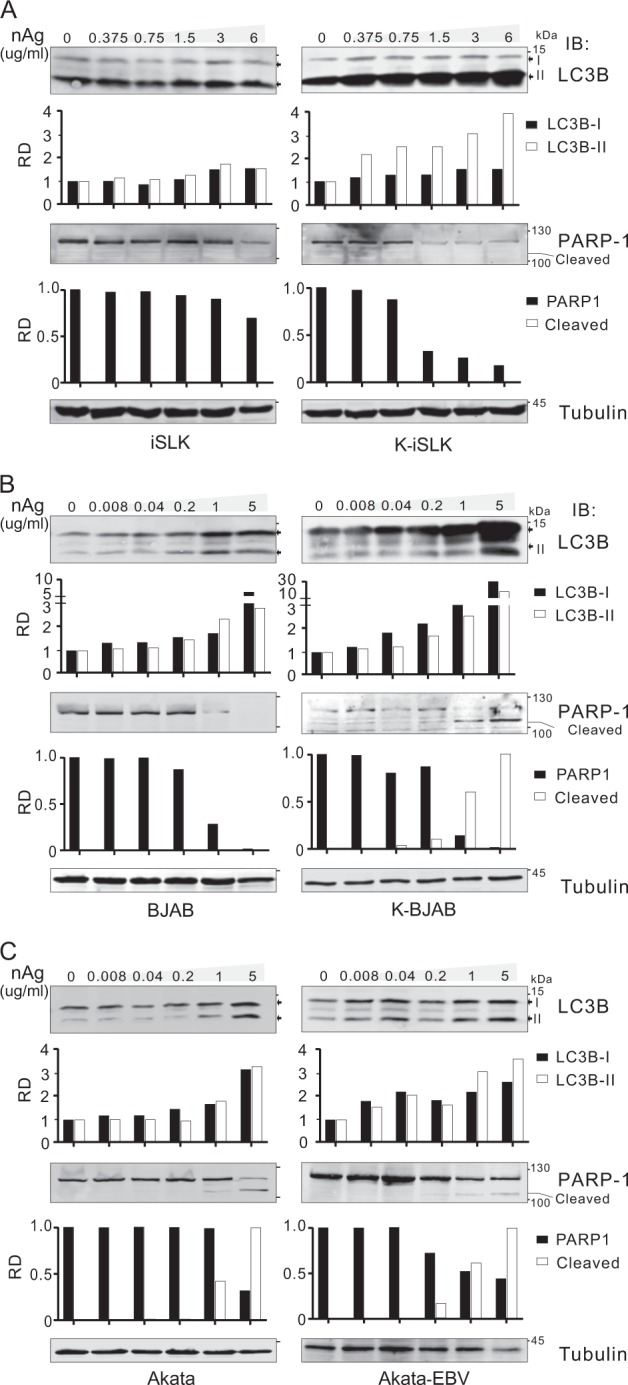
nAg induces higher activities of autophagy and apoptosis in KSHV/EBV-infected cells. Immunoblotting assays of LC3B and PARP-1 in the KSHV/EBV-infected and uninfected cells of **a** iSLK and iSLK-KSHV, **b** BJAB and K-BJAB, and **c** Akata and Akata-EBV with different dosage of 25 nm-spherical nAg treatment for 24 h. Relative density (RD) of protein bands were quantified and shown at the bottom panel. Tubulin was used as a control

The results from immunoblotting assays of LC3B and PARP-1 in both KSHV/EBV-positive and -negative tumor cells with or without nAg treatment, showed that LC3B-II accumulation and PARP-1 cleavage in the KSHV/EBV-infected tumor cells were consistently higher than that in the KSHV/EBV-uninfected tumor cells (Fig. 4a–c), indicating that nAg could induce stronger autophagy and apoptosis in KSHV/EBV-latently infected tumor cells.

### nAg induces ROS generation to activate KSHV/EBV lytic replication

**Fig. 5:**
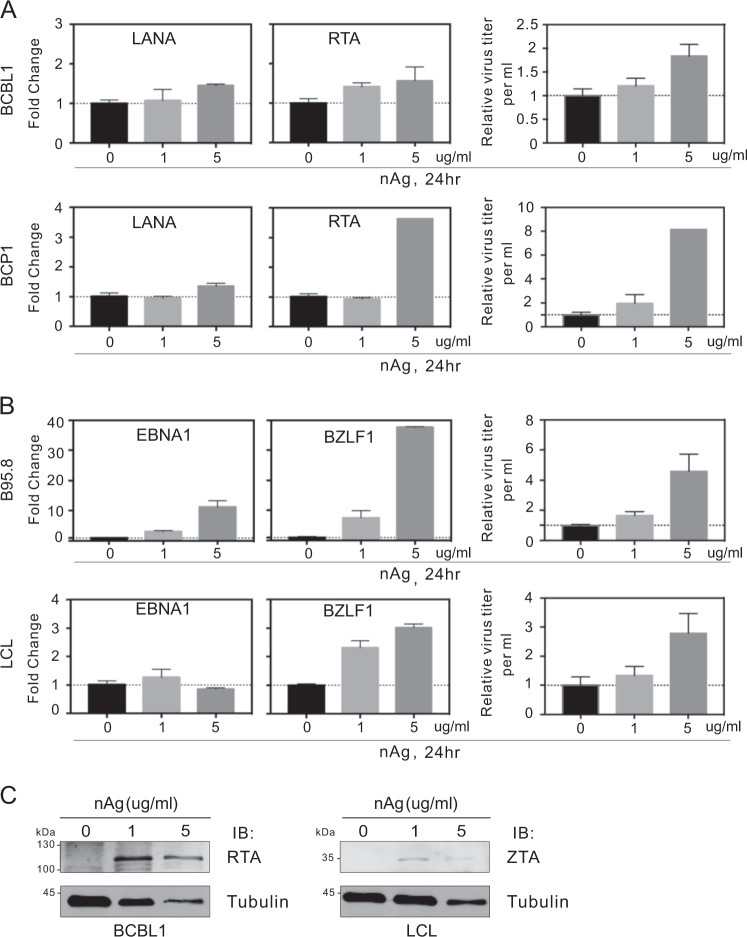
nAg activates lytic replication of KSHV/EBV-latently infected cells. Quantitative PCR analysis of **a** LANA and RTA mRNA transcripts in KSHV-latently infected cells, and **b** EBNA1 and BZLF1 mRNA transcripts in EBV-latently infected cells. The viral titers of the supernatant of cells treated with 25nm-spherical nAg shown in the left panels were individually determined and shown in the right panels. The data presented are the means ± SD from triplicate analyses. **c** Immunoblotting assays of RTA and ZTA (BZLF1) in the BCBL1/LCL cells treated with 25nm-spherical nAg from **a** and **b**. Tubulin was used as a control

Since KSHV/EBV-latently infected cells present higher sensitivity to nAg treatment than uninfected cells. To explore whether nAg also induces KSHV/EBV lytic replication from latency, we treated KSHV/EBV-latently infected B-lymphoma cells with different dosages of nAg for 24 h and individually detected the mRNA transcripts of the key latent antigen LANA/EBNA1 as well as the lytic master regulator RTA/BZLF1 within the KSHV-infected (BCBL1, BCP1) and EBV-infected (B95.8, LCL) cells, along with progeny virion production. The results showed that nAg consistently increased the transcription of RTA/BZLF1 and virion production in a dose-dependent manner, although the transcription of LANA/EBNA1 was also enhanced to some extent (Fig. 5a, b). The results of immunoblotting assays showed that the protein levels of RTA/ZTA were dramatically enhanced by nAg (Fig. 5c), strongly indicating that nAg reactivates KSHV/EBV lytic replication.

**Fig. 6:**
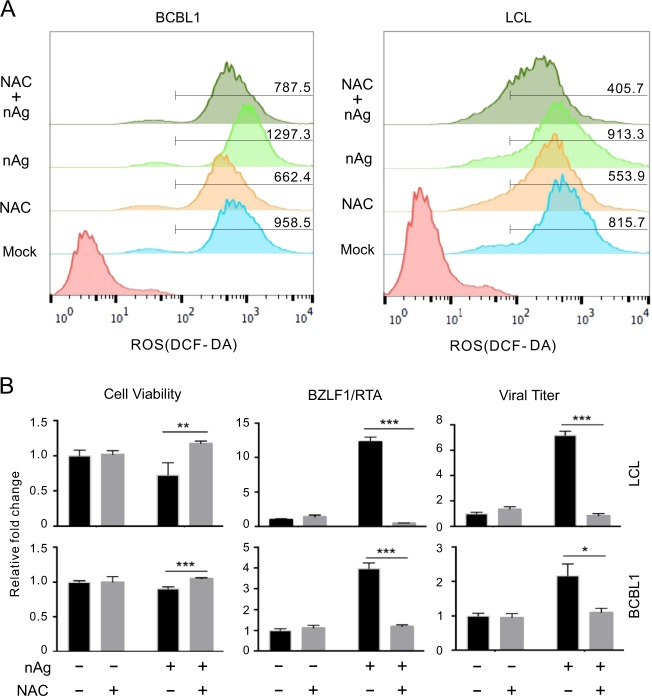
nAg enhances ROS generation to activate KSHV/EBV lytic replication. **a** nAg enhances ROS levels in BCBL1/LCL cells. Cells individually treated with NAC, 25 nm-spherical nAg (5 μg/ml) or their combination for 1 h, were subjected to flow cytometry analysis. **b** nAg-induced ROS contributes to the cytotoxicity and lytic replication of KSHV/EBV in BCBL1 and LCL cells. The cells from panel A were subjected to cell viability analysis, quantitative PCR of RTA/BZLF mRNA transcripts and viral titer of the supernatants. The data are presented as the means ± SD of triplicate analyses. Asterisks indicate *P* values as follows: *P < 0.05; **P < 0.01; ***P < 0.001

Given that nAg is able to induce cellular ROS production for cytotoxicity^27^. To determine if the life cycle switch induced by nAg is also relied on ROS generation, the cellular levels of ROS generation in KSHV/EBV-latently infected B-lymphoma cells individually treated with nAg and NAC (a ROS scavenger) combination or alone were determined by flow cytometry. The results showed that spontaneous and nAg-induced ROS were efficiently inhibited in both the KSHV-infected BCBL1 cells and EBV-infected LCL cells treated with NAC (Fig. 6a). In agreement with this phenomenon, the cytotoxicity and levels of BZLF1/RTA transcripts, as well as virion production caused by nAg in both BCBL1 and LCL cells were completely released when pretreated with NAC (Fig. 6b), indicating that ROS is indeed involved in the nAg-mediated cytotoxicity and reactivation of KSHV/EBV lytic replication.

### nAg blocks KSHV primary infection by directly destroying virion particles

**Fig. 7:**
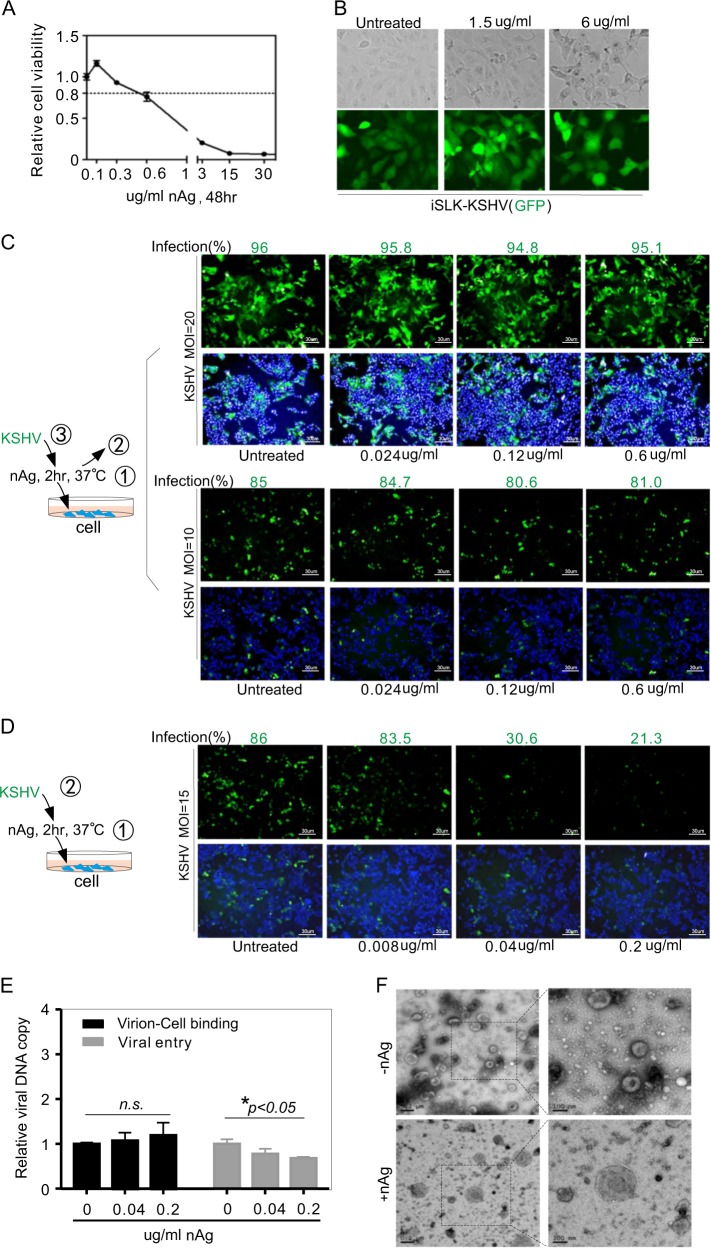
nAg inhibits KSHV primary infection via destruction of the virion particle. **a** Relative viability of HeLa cells treated with different concentrations of 25 nm-spherical nAg for 48 h. **b** nAg does not impair the immunofluorescence of GFP-tagged KSHV. Immunofluorescence assays of iSLK cells carrying GFP-tagged KSHV (iSLK-KSHV) treated with different dosage of 25 nm-spherical nAg for 48 h. Cell morphology analyzed by light microscopy was used as control. **c** The efficiency of HeLa cells with KSHV infection in the absence of nAg. Cells were incubated with different concentrations of 25 nm-spherical nAg for 2 h and then discarded before GFP-KSHV virion (generated from iSLK-KSHV cells) infection (MOI = 10, 20). A schematic diagram of the infection protocol is shown on the left panels. At 48 h post-infection, the infection percentage was quantified and shown on the top panels. **d** The infection efficiency of HeLa cells infected with KSHV virions (MOI = 15) in the presence of 25 nm-spherical nAg. A schematic diagram of the infection protocol is shown on the left panels. At 48 h post-infection, the infection percentage was quantified and shown on the top panels. **e** nAg does not impair virion-cell attachment but increase viral entry. HeLa cells incubated with KSHV virions in the presence or absence of nAg as indicated in panel D, were subjected to virion-cell attachment and viral entry analysis by quantitative PCR. Asterisk indicates *p* < 0.05. *n.s*., no significant. **f** Representative photograph of KSHV virion particle in the presence or absence of nAg under TEM. KSHV virions were incubated with or without 0.2 μg/ml 25 nm-spherical nAg for 2 h at 37 °C. Scale bars are 200 and 100 nm as indicated in the figure. Enlarged magnification is shown on the right panel

To determine if nAg also has an effect on extracellular γ-herpesvirus primary infection, HeLa cells were initially pretreated with a series of concentrations of nAg for 48 h to define the range of cell viability. The results showed that HeLa cells treated with nAg at <0.6 μg/ml presented at least 80% viability (Fig. 7a). Therefore, HeLa cells with GFP-tagged KSHV primary infection in the presence or absence of nAg were analyzed. To exclude the possibility of effect of nAg on the immunofluorescence of GFP-tagged KSHV, we have also examined the immunofluorescence intensity of GFP in the iSLK-KSHV cells carrying GFP-tagged KSHV with different dosage of nAg treatment. With the no significant effect of nAg on the immunofluorescence of GFP-tagged KSHV as parallel control (Fig. 7b), we did not observe significant inhibitory effects on HeLa cells with KSHV primary infection when nAg was replaced with different MOI solution of KSHV virion particles for 24 h (Fig. 7c). However, remarkable inhibitory effects of nAg in a dose-dependent manner were observed when nAg was maintained during infection (Fig. 7d). To elucidate the potential mechanism of the effects of nAg on KSHV primary infection, the binding ability between KSHV virions and host cell, viral entry, and transmission electron microscopy observation of negatively stained KSHV virion particles in the presence or absence of nAg was individually conducted. The results showed that nAg did not significantly impair the interaction between virion and cell, but reduced the ability of viral entry (Fig. 7e), and caused morphology change of virion particle (KSHV virions alone is a typical spherical shape with an ~100–200 nm capsid, while they became swollen and flattened after incubation with 0.2 μg/ml nAg for 2 h at 37 °C, Fig. 7f). These results indicate that nAg-mediated inhibition of viral infection is likely through direct interaction with virions.

### nAg inhibits growth of KSHV-latently infected tumor cells in vitro and in vivo

**Fig. 8:**
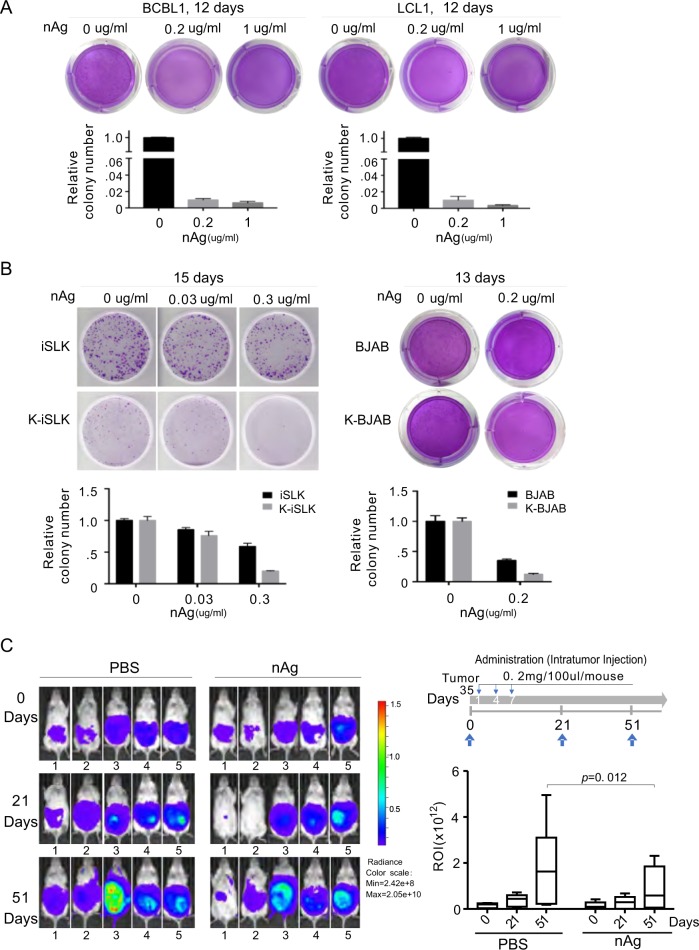
nAg exerts moderate inhibitory effects on KSHV-associated tumor growth. **a** nAg dramatically reduces colony formation of KSHV/EBV-infected cells. BCBL-1 and LCL cells were treated with different concentrations of 25 nm-spherical nAg and subjected to colony formation assays for 12 days. Bottom panel, the relative colony number was calculated from two independent experiments. **b** nAg preferentially reduces colony formation of KSHV-infected cells in vitro. KSHV-infected and uninfected iSLK and BJAB cells treated with different concentrations of 25 nm-spherical nAg were subjected to colony formation assays for 15 and 13 days. Bottom panel, the relative colony number was calculated from two independent experiments. **c** nAg moderately inhibits growth of KSHV-infected PEL in vivo. The tumor burdens of NOD/SCID mice were analyzed by luminescence assay at day 0, 21 and 51 following treatment with 25 nm-spherical nAg every two days for three times (top panel). Luminescent signals from the left panel were quantified and expressed based on region-of-interest (ROI) signal intensity (right panel). The average of luminescent signals of each group from the two experiments is shown

The observations above suggested that nAg can induce stronger cytotoxicity in KSHV/EBV-infected tumor cells and exert antiviral activity against KSHV/EBV primary infection. We next investigated whether nAg had the capability to inhibit tumor cell growth and proliferation. To accomplish this, a colony formation assay in vitro and animal experiment were conducted. After an approximately two-week culture period, the number of BCBL1 and LCL colonies was dramatically decreased by nAg when compared to the untreated control (Fig. 8a), and the inhibitory effects on colony formation of the KSHV-infected tumor cells (iSLK-KSHV and K-BJAB) were greater than those on the KSHV-uninfected tumor cells (iSLK and BJAB) (Fig. 8b). To verify the effects of nAg in the xenograft mouse model, the KSHV-associated PEL tumors in NOD/SCID mice were induced by engrafting BCBL1-Luc cells. At week 5 post-engraftment, we performed live bioluminescence imaging and selected 10 mice with higher signals for subsequent experiments. The 10 mice were randomly divided into two groups and treated with PBS or 0.2 mg nAg per mouse every 3 days for total 3 times. Bioluminescence imaging conducted at day 21 post-treatment showed that all 5 mice in the PBS group had higher luminescent signals, while the luminescent signals in mice 1 and 2 of the treatment group were dramatically reduced to almost undetectable levels, and mice 3, 4 and 5 had relatively higher initial luminescent signals. At day 51 post-treatment, the luminescent signals in mice 1 and 2 of the treatment group increased, while those of other mice remained elevated. Comparison of the average fold change of luminescent signals of mice from the two groups revealed a moderate decrease in fold change for mice treated with nAg relative to mice treated with PBS (Fig. 8c). These results demonstrated that nAg had moderate inhibitory effects on tumor development, and the effects were better for mice with smaller tumors.

## Discusion

**Fig. 9:**
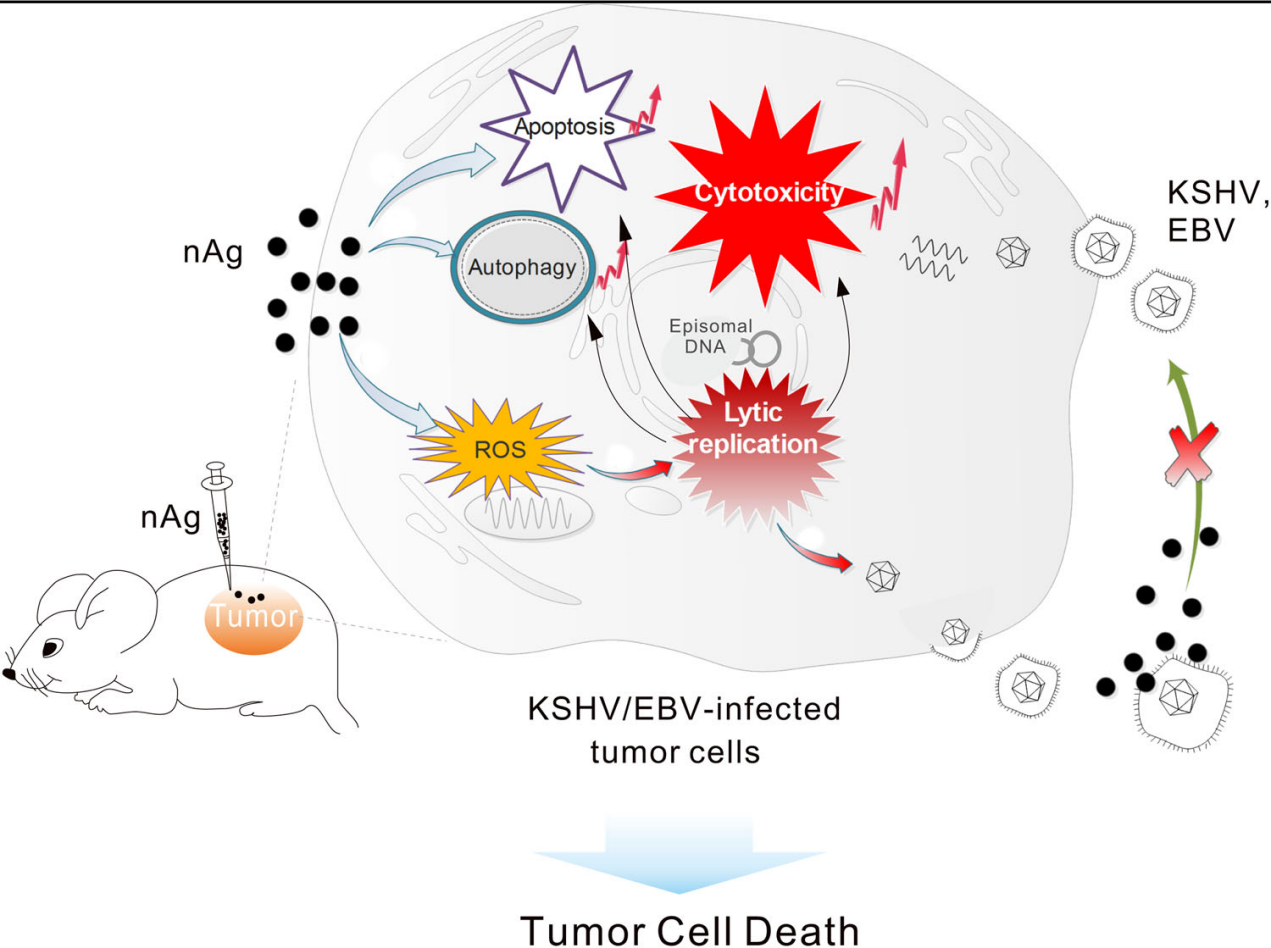
Proposed model of nAg-mediated inhibitory effects on γ-herpesvirus-associated tumor cells. nAg exhibits stronger cytotoxicity in the γ-herpesvirus-infected tumor cells through inducing viral lytic reactivation, which relies on ROS generation. Moreover, nAg can block further primary infection by directly destroying virion particles

The oncogenic γ-herpesviruses KSHV and EBV are causative agents of many human malignancies that are generally resistant to conventional chemotherapy. The occurrence of KSHV/EBV-associated tumors is closely associated with viral latency, and manipulation of host genes by the viral latent genes usually contributes to the tumorigenesis process. The viruses themselves can serve as potential targets for therapy because almost all tumor cells carry them. Therefore, a common strategy for KSHV/EBV-associated tumor treatment is to promote virus entry into lytic replication from latency. When this occurs, the quiescent condition is broken, after which a large number of lytic genes are expressed following virion packaging and release, leading to death of the tumor cell. However, virions released during lytic replication may lead to new infection and increase the long-term risk associated with tumors. In this study, we found for the first time that nAg presented exclusively higher cytotoxicity on KSHV/EBV-latently infected cells through reactivating viral lytic replication, and that this also blocks KSHV primary infection by directly destroying virion particles (Fig. 9). These findings indicate that nAg could be a promising therapeutic agent for treatment of KSHV/EBV-associated tumors by targeting latent and lytic replication of the life cycle. In agreement with previous findings about the effect of nAg^26^, activation of autophagy and apoptosis were observed in KSHV/EBV-latently infected cells that received nAg treatment.

nAg toxicity usually depends on properties such as size, shape and surface coating^28^. Smaller sized nAg are taken up into cells in greater abundance through endocytosis, leading to greater toxicity^9,29^, while larger nAg are not internalized^30,31^. Consistent with previous reports^24,28^, we observed that the shape of nAg also influences their cytotoxicity toward epithelial and B-lymphoma cells, with smaller spherical nAg having higher toxicity than larger spherical nAg.

It has been demonstrated that viral lytic replication can be induced by a variety of extracellular stresses, such as cytokine signaling, cell differentiation, co-infection, hypoxia^20,32^. nAg not only induces KSHV/EBV-latently infected tumor cell cytotoxicity in a dose-dependent and time-dependent manner, it also has greater toxicity toward KSHV/EBV-infected cells than toward uninfected tumor cells. Our studies revealed that nAg-induced cytotoxicity and lytic replication can be blocked when pretreated with the effective ROS scavenger NAC, suggesting that ROS is involved in nAg-mediated lytic reactivation and fundamental cytotoxicity, and that nAg is another stimuli to reactivate lytic replication from latency. Based on the finding that nAg presents specific greater cytotoxicity toward tumor cells harboring KSHV/EBV, we could adjust the treatment concentration and time of nAg to efficiently control the development of KSHV/EBV-associated tumors. More importantly, we found that nAg could directly destroy the KSHV virion particles and block viral primary infection, even at concentrations that are not cytotoxic. Despite previous studies have shown that nAg can preferentially bind with the gp120 subunit of viral envelope glycoprotein to block HIV-1 CD4-dependent virion entry^3^, the entry of γ-herpesvirus into host cells relies on several corresponding cellular receptors^33,34^, the specific cellular receptor or corresponding viral glycoprotein interaction with nAg for blocking KSHV primary infection requires further investigation. In addition, although the treatment of nAg did not dramatically impair the interaction of viron and cell, the finding of increase of viral entry indicate that nAg treatment may enhance the efficiency of viral entry of permissive cell surfaces, and then influence KSHV primary infection for latent-infection establishment at long-term time point.

During treatment of γ-herpesvirus associated diseases, it is often ignored the fact that co-infection is a common phenomenon for γ-herpesvirus. For instance, populations affected by KSHV are usually composed of individuals with immunodeficiency, such as AIDS patients^35^. EBV, which occurs as an asymptomatic life-long infection in ~95% of the world’s population, can also co-infect with HIV and several other viruses, such as HCV and hCMV^36^. Our studies provide evidence that nAg has also an antiviral activity against KSHV and EBV, which added up broad-spectrum antiviral activities against co-infection of both KSHV/EBV and different viruses including drug-resistant strain HIV. Therefore, nAg has greater advantages than conventional drugs because it has a broad-spectrum and does not induce drug-tolerance.

In addition, in view of the inhibitory effect of nAg on KSHV/EBV-associated tumors, although it exerts significant inhibitory effects on the colony formation of KSHV/EBV-infected cells in vitro, only a moderate inhibition of tumor growth was observed in vivo in a xenograft mouse model. This could be due to only small amounts of total nAg are accumulated in the target tissues after injection into body. To avoid this problem, nAg can be linked with specific polypeptides targeting tumor cells or cellular receptors of virus entry to increase the efficiency of specific targeting for tumor cells or viruses. Based on advanced nanotechnology, we believe next generation of nAg can be refined to impart it with addition functions.

## Materials and methods

### Characterization of nAg

Silver nanoparticles (nAg) with different size and shape, which generated by using liquid-chemical synthesis technology and polyvinylpyrrolidone (PVP) as a coating buffer, were purchased from Shanghai Huzheng Nanotechnology Co. Ltd., CN, or nanoComposix, Inc., USA. Stock nAg solutions were stored at 4 °C in the dark.

### Ethics statement and husbandry

Healthy human blood was obtained from Shanghai Skin Disease Hospital. Usage of blood samples for research purposes was approved by the Hospital Medical Ethics Committee. All of the animal studies were conducted in accordance with the Chinese Guide for the Care and Use of Laboratory Animals. All of the experiments were approved and overseen by the institutional animal care and use committee of Fudan University under Protocol ID 196086. Mice were housed in microisolator cages with no more than 5 mice per cage in certified specific-pathogen-free or germ-free vivaria. Animals were provided with access to autoclaved water and food ad libitum.

### Reagents and antibodies

Antibodies to PARP1 (F2, Santa cruz), Caspase-3 (H-277, Santa cruz), EBV ZEBRA (sc-53904, Santa cruz), LC3B (GT3612, GeneTex), SQSTM1 (ab56416, Abcam) and Tubulin (66031-1, Proteintech) were used according to the manufacturers specifications. Mouse monoclonal antibodies against RTA were kindly provided by Ke Lan from Wuhan University. Tetradecanoyl Phorbol Acetate (TPA) was purchased from Sigma and sodium butyrate from J&K Corporation. 3-Methyladenine (3-MA), and Choloquine (Chl) were purchased from Sigma-Aldrich. Doxycycline (DOX) was purchased from Sangon Biotech (Shanghai).

### Cells cultures

The EBV and KSHV negative (HEK 293T, iSLK, HeLa, BJAB, and Akata), KSHV-positive (iSLK-KSHV, K-HeLa, K-BJAB, BCBL-1, and BCP1), and EBV-positive (Akata-EBV, B95.8, LCL) cell lines were used. Human peripheral blood mononuclear cells (PBMCs) were used as primary cell lines. Both iSLK and iSLK-KSHV cells were gifts from Shou-Jiang Gao at the University of South California, PBMC were isolated from healthy human blood (from Shanghai Skin Disease Hospital), and other cell lines were from the American Type Culture Collection (ATCC). Adherent cells and suspension cells were maintained in Dulbecco’s modified Eagle’s medium (DMEM) and Roswell Park Memorial Institute (RPMI 1640) medium, respectively, containing 10% fetal bovine serum (FBS) and 1% penicillin and streptomycin. iSLK were grown in the presence of 1 mg/ml hygromycin and 250 μg/ml G418, while iSLK-Bac16 grew in the presence of 1 mg/ml hygromycin, 250 μg/ml G418, and 1 μg/ml puromycin. All of the cell lines were incubated at 37 °C in a 5% CO_2_ incubator, during which time they were passaged once every 2 to 3 days. DMEM, RPMI-1640, FBS and Trypsin were purchased from Gibco (USA).

### Cytotoxicity assay

The cytotoxicity of nAg treatments towards adherent cells was determined by methyl thiazolyl tetrazolium (MTT, Sigma-Aldrich, St. Louis, MO) assay. The adherent cells were seeded into a 96-well plate at a density of 7 × 10^3^/100 μl and held at 37 °C in a 5% CO_2_ incubator overnight, after which the media was replaced with 100 μl medium containing nAg (five replicates) and further incubated for 24 h and 48 h. Following treatment, MTT solution was added and incubated for 4 h at 37 °C. After discarding the medium, 150 μl MTT Solvent [4 mM HCl, 0.1% Nondet P-40 (NP40) in isopropanol] was added for solubilization and the absorbance value was measured. The cytotoxicity of nAg treatments towards cell suspensions was determined by trypan blue (Sigma-Aldrich, St. Louis, MO, USA) assay. The cell suspensions were incubated with nAg at various dilutions (five replicates) for 24 h and 48 h. The amount and proportion of living cells were determined using cell viability image analyzer Vi-CELL XR (Beckman Coulter, Indianapolis, IN, USA) and the results were normalized against the viability of the control. The 50% cytotoxic concentration (CC50) was calculated from the following equation: log (inhibitor) vs response curve, which was given by Y = bottom + (top−bottom)/(1 + 10(Log IC50−X)×slope) using the GraphPad Prism 7 program.

The morphology of cells incubated with nAg was visualized using a standard microscope or a Leica SP8 confocal microscope after TRITC phalloidin (YEASEN #40734ES75, CN) staining according to the manufacturer’s instructions. Nuclei were counterstained with 4, 6-diamidino-2-phenylindole (DAPI). Briefly, cells that were treated with nAg were washed with PBS twice, then fixed in 4% paraformaldehyde for 20 min, after which cell suspensions were allowed to attach to the plate. The cells fixed on the plate were permeabilized in PBS containing 0.2% fish skin gelatin and 0.2% Triton X-100 for 5 min, then incubated with 100 nM TRITC Phalloidin for 30 min at room temperature.

### Apoptosis analysis

Intracellular apoptosis levels were detected by Annexin-V and propidium iodide (PI) staining using an Annexin V FITC Apoptosis Kit (BD #556547, USA), according to the manufacturer’s instructions with a flow cytometry. Briefly, 1 × 10^6^ BCBL1 cells were collected and washed with cold PBS twice after treatment with nAg for 48 h, then suspended with 100 μl binding buffer, and incubated with Annexin V FITC and PI at room temperature for 15 min. Cellular apoptosis level was measured with a FACScan flow cytometer and analyzed with the FlowJo software. For each sample 10,000 events were collected.

### Detection of Intracellular ROS

Intracellular ROS levels were measured using an oxidation-sensitive fluorescent probe (DCFH-DA) through flow cytometry according to the manufacturer’s instructions of Reactive Oxygen Species Assay Kit (Beyotime Co., CN). Briefly, 1 × 10^6^ cells were collected and washed with PBS twice after treatment with nAg for 2 h in the presence or absence of 10 mM N-acetyl-L-cysteine (NAC, Beyotime Co., CN) for 1 h, then incubated with 10 μM DCFH-DH at 37 °C for 20 min. Specifically, DCFH-DA was deacetylated into DCFH using intracellular nonspecific esterase, then further oxidized by intracellular ROS into DCF with fluorescence, which was detected using a FACScan flow cytometer (BD Biosciences, Foster, CA, USA) and analyzed with the FlowJo software. For each sample 10,000 events were collected.

### Quantitative PCR

Total RNA was extracted by using TRIzol reagent (Invitrogen, US) according to manufacturer’s instruction, and reverse transcribed into cDNA with Superscript II reverse transcription kit (YEASEN, CN). The cDNA was amplified in a 20 μl total volume containing 10 μl SYBR green, 0.4 μl each primer (10 μM), 4.2ul H_2_O, and 5 μl cDNA. A melting-curve analysis was performed to verify the specificities of the amplified products. The values for the relative levels of change were calculated by the threshold cycle (ΔΔCT) method, and samples were tested in triplicates. The primers targeting KSHV ORF73 (sense: 5′-GGGGTACACACTACGGTTGG-3′, anti-sense: 5′-TCCCGCAACACCT TTACCTC-3′, 429 bps), KSHV ORF50 (sense: 5′-GATGTCGGGTCGCCTCTCTC-3′, anti-sense: 5′-GTGCCGGACTCCTGTACCTC-3′, 228 bps), EBV EBNA1 (sense: 5′-CTGGAAATGGCCTAGGAGAG-3′; anti-sense: 5′-TATGTCTTGGCCCTGATCCT-3′, 171 bps), and EBV BZLF1 (sense: 5′-TGATTCTGGGTTATGTCGGA-3′; anti-sense: 5′-AGGCCAGCTAACTGCCTATC-3′, 137 bps), respectively. The ORF72 (sense: 5′-TATTTGGGACCTTTCAACAATCTCTT-3′; anti-sense: 5′- GTTCCACTGCCGCCTGTA-3′, 609 bps) or EBNA1 (sense: 5′- CAATGGTGTAAGACGACATT -3′; anti-sense: 5′- CCTGTAGGGGAAGCCGAT -3′, 387 bps) were used for quantitation of KSHV and EBV viral titer, respectively. Actin (sense: 5′-GGCATCCACGAAACTACCTT-3′; anti-sense: 5′- TGATCTCCTTCTGCATCCTG -3′, 136 bps) was used as an internal control.

### Protein Extraction and Immunoblotting

The harvested cells were washed twice with cold PBS, then lysed in 500 μl RIPA buffer [150 mM NaCl, 50 mM Tris (pH 7.6), 1% Nonidet P-40, 2 mM EDTA, proteinase inhibitor mixture (1 mM phenmethyl sulfonyl fluorine, 1 g/ml aprotinin, 1 g/ml leupeptin, and 1 g/ml pepstatin)] for 30 min with constant oscillation at 4 °C. After centrifugation at 14,500 rpm, 4 °C for 5 min, cell debris was removed. The supernatant was then transferred to a new eppendorf tube, after which the protein samples were boiled in 6x DTT loading buffer according to the concentration of protein that was determined by the Bradford method. Next, the samples were separated by SDS-PAGE and then transferred to a 0.45-mm nitrocellulose filter. After blocking, the protein in the membrane was probed with primary antibodies at 4 °C overnight, and then incubated with appropriate secondary antibodies for another 1 h at room temperature. Finally, the membrane was visualized using an Odyssey Infrared scanner (Li-Cor Biosciences, US) for optical density analysis.

### Extraction and quantitation of virion production

The supernatant of KSHV/EBV-positive cell lines treated with nAg was harvested and digested with DNase at 37 °C for 30 min to remove cellular DNA debris, after which the reaction was terminated by treatment with 5 mM EDTA at 75 °C for 10 min. Samples were then resuspended in HMW buffer, 0.5% SDS and 100 μg/ml proteinase K at 60 °C for 2 h. Next, an equal volume of phenol and chloroform (1:1) was added, after which the samples were centrifuged at 12,000 rpm, 4 °C for 5 min. Episomal DNA in the supernatant was subsequently precipitated by adding 3 M sodium acetate (1/10, v/v) and twice the volume of 100% alcohol, then stored at −80 °C for 30 min, after which samples were centrifuged at 12,000 rpm, 4 °C for 10 min. Finally, the DNA was washed, dried in the air, dissolved in deionized water, and used for virion quantification by qPCR.

### KSHV Virion Purification and Primary Infection

iSLK-KSHV cells carrying GFP-tagged KSHV genome as described previously^37^, were induced with 1 μg/ml of Dox and 1 mM sodium butyrate (NaB) for 5 days at 37 °C under 5% CO_2_. Following induction, the supernatant of the culture medium was filtered through a 0.45 μm filter on ice, after which the viral particles were spun down at 25,000 rpm for 2 h at 4 °C. Finally, the concentrated viruses were then collected and re-suspended in RPMI 1640 medium, and stored at −80 °C until used for virion quantification by qPCR.

HeLa cells were seeded into 96-well plates at a density of 1 × 10^4^ cells per well containing 100 μl media overnight to adhere, then exposed to nAg at different final concentrations and further incubated for 2 h at 37 °C. The media containing nAg was discarded or maintained during infection. After which KSHV extracted from iSLK-KSHV cells was added at a MOI (multiplicity of infection) of 10 to 20, then spin infected (1500 g, 25 °C) for 1 h and finally incubated at 37 °C for 48 h. KSHV *de novo* infection of HeLa cells was performed through spin infection and the infection efficiency was determined by counting the percentage of GFP-positive cells after infection.

### Virion-cell binding and viral entry assay

To determine virion-cell attachment, 1 × 10^5^ HeLa cells that treated with different dosage of nAg (0, 0.04, 0.2 μg/ml) at 37 °C for 2 h were incubated with KSHV on ice for 1 h. Then cells were washed twice with PBS to remove unbound virus and lysed immediately by DNA extraction. For viral entry, HeLa cells that incubated with KSHV on ice for 1 h, were followed by incubation for another 1 h at 37 °C. Then cells were digested with 0.05% trypsin-EDTA at 37 °C for 5 min to remove non-internalized virus. Finally, the cells were washed twice with PBS and lysed immediately by DNA extraction. For both viral-cell binding and entry assays, KSHV DNA copies in cells were quantitated by qPCR using ORF73 as target.

### Transmission Electron Microscopy (TEM)

KSHV virion (concentration was 2 × 10^8^/ml, 20 μl) was mixed with or without nAg at final concentration of 0.2 μg/ml and maintained at 37 °C for 2 h. A copper mesh with carbon support film was placed over the sample droplets (50 μl sample containing 10^7^/ml of virus particles) and allowed to float for 3–10 min to ensure that the virus particles could adsorb onto the support film. The copper mesh was then removed from the sample droplets and placed on filter paper for liquid absorption, followed by staining on the 2% phosphotungstic acid-dyed droplets, and floating for 3 min. After absorbing the liquid, the copper mesh was dried under an incandescent lamp for 10 min and then observed under a FEI Tecnai G2 Spirit transmission electron microscopy (Thermo Fisher Scientific, USA).

### Colony formation assay

Adherent cells were cultured in 10 cm dishes (3000 cells/plate) for 24 h to adhere, after which culture was continued in the presence of nAg for 15 days. Next, the medium was removed, and cells were washed twice with PBS, fixed with 4% formaldehyde and stained with 0.1% crystal violet. Colony formation in each dish was scanned using a Li-Cor Odyssey image system.

Soft-agar colony formation assay was used to generate cell suspensions. Briefly, two soft agar layers were placed into 6-well plates. The base agar layer consisted of 2 ml DMEM containing 10% FBS and 0.75% agar that was melted in a microwave and then kept at 56 °C, while the top agar layer consisted of 2 × 10^4^ cells in 2 ml DMEM containing 10% FBS, 0.36% agar and nAg at different final concentrations. After about 2 weeks, cells were stained with 0.04% crystal violet and 2% ethyl alcohol and colony formation in each dish was photographed.

### Animal experiments

Five-week-old female NOD/SCID mice (purchased from Beijing Vital River Laboratory Animal Technology Co., Ltd., Beijing, CN) were injected intraperitoneally with 200 μl of PBS containing 10 × 10^6^ BCBL1-Luc cells. The mice were then subjected to live imaging at 5 weeks post-inoculation. Briefly, mice were injected with D-luciferin at 150 mg/kg body weight twelve minutes later, the mice were imaged for 0.1 s, 0.5 s using an IVIS Spectrum Imaging System (PerkinElmer, USA). Ten successfully established model mice were randomly divided into two groups, a treatment group that received intraperitoneal injection of 100 μl nAg at 2 mg/ml every two days until three injections had been administered, and a control that received intraperitoneal injections of PBS under the same conditions. At day 21 and 51 post-treatment, tumor development in the model mice was observed via live images and the results were presented as total radiance within the ROI after mice were imaged for 0.5 s.

### Statistical analysis

Statistical parameters including the definition and exact values of *n*, distribution and deviation are reported in the figure legends. Data are expressed as mean±standard deviation (SD). The significance of the variability between two groups was determined by one-way analyses of variance using GraphPad Prism 7 software (GraphPad Software, Inc, La Jolla, CA, USA). A *p* value of <0.05 was considered statistically significant and a *p* value of >0.05 was considered statistically non-significant.

## References

[1] Galdiero S (2011). Silver nanoparticles as potential antiviral agents. Molecules.

[2] Sun R. W., et al. Silver nanoparticles fabricated in Hepes buffer exhibit cytoprotective activities toward HIV-1 infected cells. *Chem. Commun. (Camb)* **40**, 5059–5061 (2005). 10.1039/b510984a 16220170

[3] Lara HH, Ayala-Nunez NV, Ixtepan-Turrent L, Rodriguez-Padilla C (2010). Mode of antiviral action of silver nanoparticles against HIV-1. J. Nanobiotechnol..

[4] Lara HH, Ixtepan-Turrent L, Garza-Trevino EN, Rodriguez-Padilla C (2010). PVP-coated silver nanoparticles block the transmission of cell-free and cell-associated HIV-1 in human cervical culture. J Nanobiotechnol..

[5] Lu L (2008). Silver nanoparticles inhibit hepatitis B virus replication. Antivir. Ther..

[6] Baram-Pinto D, Shukla S, Gedanken A, Sarid R (2010). Inhibition of HSV-1 attachment, entry, and cell-to-cell spread by functionalized multivalent gold nanoparticles. Small.

[7] Baram-Pinto D, Shukla S, Perkas N, Gedanken A, Sarid R (2009). Inhibition of herpes simplex virus type 1 infection by silver nanoparticles capped with mercaptoethane sulfonate. Bioconjug. Chem..

[8] Papp I (2010). Inhibition of influenza virus infection by multivalent sialic-acid-functionalized gold nanoparticles. Small.

[9] AshaRani PV, Low Kah Mun G, Hande MP, Valiyaveettil S (2009). Cytotoxicity and genotoxicity of silver nanoparticles in human cells. ACS Nano.

[10] Nallathamby PD, Xu XH (2010). Study of cytotoxic and therapeutic effects of stable and purified silver nanoparticles on tumor cells. Nanoscale.

[11] Zhang X. F., Liu Z. G., Shen W. & Gurunathan S. Silver nanoparticles: synthesis, characterization, properties, applications, and therapeutic approaches. *Int. J. Mol. Sci.* **17**, e1534 (2016). 10.3390/ijms17091534 PMC5037809 27649147

[12] Gurunathan S (2009). Antiangiogenic properties of silver nanoparticles. Biomaterials.

[13] Kemp MM (2009). Gold and silver nanoparticles conjugated with heparin derivative possess anti-angiogenesis properties. Nanotechnology.

[14] Liu J (2012). TAT-modified nanosilver for combating multidrug-resistant cancer. Biomaterials.

[15] Sriram MI, Kanth SB, Kalishwaralal K, Gurunathan S (2010). Antitumor activity of silver nanoparticles in Dalton’s lymphoma ascites tumor model. Int. J. Nanomed..

[16] Saha A, Kaul R, Murakami M, Robertson ES (2010). Tumor viruses and cancer biology Modulating signaling pathways for therapeutic intervention. Cancer Biol. Therapy.

[17] de Martel C (2012). Global burden of cancers attributable to infections in 2008: a review and synthetic analysis. Lancet Oncol..

[18] Young LS, Yap LF, Murray PG (2016). Epstein-Barr virus: more than 50 years old and still providing surprises. Nat. Rev. Cancer.

[19] Reid EG (2011). Bortezomib-induced Epstein-Barr virus and Kaposi sarcoma herpesvirus lytic gene expression: oncolytic strategies. Curr. Opin. Oncol..

[20] Giffin L, Damania B (2014). KSHV: pathways to tumorigenesis and persistent infection. Adv. Virus Res..

[21] Dzeng RK (2015). Small molecule growth inhibitors of human oncogenic gammaherpesvirus infected B-cells. Mol. Oncol..

[22] Andrei G, Snoeck R (2015). Kaposi’s sarcoma-associated herpesvirus: the role of lytic replication in targeted therapy. Curr. Opin. Infect. Dis..

[23] Pratsinis A, Hervella P, Leroux JC, Pratsinis SE, Sotiriou GA (2013). Toxicity of silver nanoparticles in macrophages. Small.

[24] George S (2012). Surface defects on plate-shaped silver nanoparticles contribute to its hazard potential in a fish gill cell line and zebrafish embryos. ACS Nano.

[25] Mao BH, Tsai JC, Chen CW, Yan SJ, Wang YJ (2016). Mechanisms of silver nanoparticle-induced toxicity and important role of autophagy. Nanotoxicology.

[26] Lee YH (2014). Cytotoxicity, oxidative stress, apoptosis and the autophagic effects of silver nanoparticles in mouse embryonic fibroblasts. Biomaterials.

[27] Avalos A, Haza AI, Mateo D, Morales P (2014). Cytotoxicity and ROS production of manufactured silver nanoparticles of different sizes in hepatoma and leukemia cells. J. Appl. Toxicol..

[28] Wang Z, Xia T, Liu S (2015). Mechanisms of nanosilver-induced toxicological effects: more attention should be paid to its sublethal effects. Nanoscale.

[29] Asharani PV, Hande MP, Valiyaveettil S (2009). Anti-proliferative activity of silver nanoparticles. BMC Cell Biol..

[30] Verano-Braga T (2014). Insights into the cellular response triggered by silver nanoparticles using quantitative proteomics. ACS Nano.

[31] Miethling-Graff R (2014). Exposure to silver nanoparticles induces size- and dose-dependent oxidative stress and cytotoxicity in human colon carcinoma cells. Toxicol..

[32] Purushothaman P, Uppal T, Verma SC (2015). Molecular biology of KSHV lytic reactivation. Viruses.

[33] Gillet L, Frederico B, Stevenson PG (2015). Host entry by gamma-herpesviruses–lessons from animal viruses?. Curr. Opin. Virol..

[34] Veettil MV, Bandyopadhyay C, Dutta D, Chandran B (2014). Interaction of KSHV with host cell surface receptors and cell entry. Viruses.

[35] da Silva APF (2015). Human gammaherpesviruses viraemia in HIV infected patients. J. Clin. Pathol..

[36] Saghafian-Hedengren S (2013). Epstein-Barr virus coinfection in children boosts cytomegalovirus-induced differentiation of natural killer cells. J. Virol..

[37] Brulois KF (2012). Construction and manipulation of a new Kaposi’s sarcoma-associated herpesvirus bacterial artificial chromosome clone. J. Virol..

